# Analysis of lethal and sublethal impacts of environmental disasters on sperm whales using stochastic modeling

**DOI:** 10.1007/s10646-017-1813-4

**Published:** 2017-05-12

**Authors:** Azmy S. Ackleh, Ross A. Chiquet, Baoling Ma, Tingting Tang, Hal Caswell, Amy Veprauskas, Natalia Sidorovskaia

**Affiliations:** 1 Department of Mathematics, University of Louisiana at Lafayette, Lafayette, LA 70504-1010 USA; 2 Department of Mathematics, Millersville University, Millersville, PA 17551-0302 USA; 3 Department of Biology, Woods Hole Oceanographic Institution, Woods Hole, MA 02543 USA; 4 Institute for Biodiversity and Ecosystem Dynamics, University of Amsterdam, Amsterdam, The Netherlands; 5 Department of Physics, University of Louisiana at Lafayette, Lafayette, LA 70504-1010 USA

**Keywords:** Population recovery, Environmental disasters, Stochastic modeling, Lethal impact, Sublethal impact, Sperm whales

## Abstract

Mathematical models are essential for combining data from multiple sources to quantify population endpoints. This is especially true for species, such as marine mammals, for which data on vital rates are difficult to obtain. Since the effects of an environmental disaster are not fixed, we develop time-varying (nonautonomous) matrix population models that account for the eventual recovery of the environment to the pre-disaster state. We use these models to investigate how lethal and sublethal impacts (in the form of reductions in the survival and fecundity, respectively) affect the population’s recovery process. We explore two scenarios of the environmental recovery process and include the effect of demographic stochasticity. Our results provide insights into the relationship between the magnitude of the disaster, the duration of the disaster, and the probability that the population recovers to pre-disaster levels or a biologically relevant threshold level. To illustrate this modeling methodology, we provide an application to a sperm whale population. This application was motivated by the 2010 Deepwater Horizon oil rig explosion in the Gulf of Mexico that has impacted a wide variety of species populations including oysters, fish, corals, and whales.

## Introduction

A disturbance, natural or anthropogenic, that causes a sufficiently great reduction in the vital rates will cause a growing population to decline. In general, we can expect the effects of a disturbance to gradually diminish over time, eventually leading to a return to positive population growth. The recovery of a population following such a disturbance is determined by the vital rates (survival, development, fecundity) of the species under consideration as well as how those rates change over the recovery period. Matrix population models can be useful tools for studying the potential impact of a disturbance on the population recovery process.

We develop nonautonomous (vital rates depending explicitly on time) matrix population models to account for environmental changes from a disturbance. To investigate the long term effect of a disturbance on a population, we incorporate time dependent environmental recovery functions into a matrix population model. These recovery functions take into account the impact of the disturbance on the vital rates, how long the disturbance affects the population, and the time required for the environment to permit the vital rates to return to pre-event levels. The environmental recovery process appears in the matrix population model in the form of a time course of reductions in survival rates or fecundity. These reductions are assumed to represent the lethal and sublethal impacts of a disturbance, respectively. The resulting time varying matrix models can be used to assess population endpoints. Here, we examine a population’s recovery process by calculating the mean time to recovery or recovery probabilities, where recovery is defined to be the return to either the pre-event population size or a biologically relevant threshold value.

To illustrate this modeling methodology, we provide an application to a sperm whale population. The sperm whale, *Physeter macrocephalus*, is one of the most ecologically important mammals in the ocean (Whitehead 2003). However, their population is very fragile as noted in studies by Chiquet et al. (2013), Whitehead (2003), and Whitehead and Gero (2015) and has been shown to be growing at a slow rate (Chiquet et al. 2013; Whitehead 2003). Hence, the population is susceptible to many natural and man-made threats (Carrillo and Ritter 2010; Haase and Félix 1994; Laist et al. 2001; Laist 1997; Di Natale and Notarbartolo di Sciara 1994; Whitehead 2003).

Our particular interest is in sperm whales in the northern Gulf of Mexico (GoM). Abundance estimates from 1991 to 2009 suggest that, at most, there are 1665 sperm whales in the northern GoM (Waring et al. 2012). In general, movement out of the GoM by females and juveniles does not occur (Waring et al. 2012). Consequently, GoM sperm whales are considered to be distinct from sperm whales in the Atlantic Ocean. On average, GoM sperm whales are smaller in size and the group size of females and immature whales is about one-third the size of populations found in other areas. There are also significant genetic differences between sperm whales in the GoM compared to those in the North Atlantic Ocean (Jochens et al. 2008; Waring et al. 2012). Since the GoM sperm whale population is small, closed, and slowly growing, population viability may be significantly impacted by a disturbance that reduces vital rates.

The Deepwater Horizon (DWH) oil rig explosion in April of 2010 is the type of event that could have such an impact on the sperm whale population in the Northern Gulf of Mexico (GoM). The DWH explosion caused the largest oil spill in US waters and is one of the worst environmental disasters in US history (Barlow et al. 1995; Levy and Gopalakrishnan 2010; Ramseur 2010). It is not known how long GoM sperm whales were exposed to toxicants from the spill or whether they relocated as a result of the spill (Merkens et al. 2013; Ackleh et al. 2012). However, acoustic studies from 2007 and September 2010 confirm that sperm whales were present in areas impacted by the spill (Merkens et al. 2013; Ackleh et al. 2012).

The effects of oil spills on sperm whale populations have not been studied enough to determine their long term lethal and sublethal impacts on the population or how these effects impact population recovery. Nor are there any estimates of sperm whale vital rates specific to the Gulf of Mexico. This situation is the rule rather than the exception in conservation biology, because demographic rates have been estimated for only a small minority of species, and toxicant effects have been measured for an even smaller subset. Therefore, given this lack of data, it is an established practice to use approximate demographic information, from other populations or other species (e.g., Caswell et al. (1998) for bycatch mortality in harbour porpoise). Banks et al. (2014) have discussed the general issues involved with such extrapolation. Combining these approximate analyses with a reasonable extrapolation of toxicant effects measured in other species can provide a general picture for effects, can provide a baseline for comparison if data do become available, and can be generalized to other species with similar life histories. This is our goal for sperm whales in the Gulf of Mexico exposed to oil pollution.

Toxic physiological effects of the oil spill have been documented. Direct exposure to the vapors released from oil are assumed to cause soft tissue irritation (Geraci and Aubin 1990). Meanwhile, Wise et al. (2014a) showed that the dispersants Corexit 9527 and 9500 are both cytotoxic to sperm whale skin fibroblasts. In addition, they found that Corexit 9527 is genotoxic which could affect calf development or result in loss of offspring (Wise et al. 2014a, b). The inhalation or digestion of the oil, vapors, or dispersants are also assumed to impact the respiratory and gastrointestinal tract. This damage can lead to pneumonia and digestive problems, and may eventually increase mortality (Waring et al. 2012; Geraci and Aubin 1990).

Studies of other cetacean species also provide evidence that the toxicants released during an oil spill can impact the survival rates and fecundity of marine mammals. For instance, oil spills have been shown to have lethal effects on killer whales (Matkin et al. 2008) and sublethal effects on bottlenose dolphins (Lane et al. 2015). Following the DWH oil spill, Lane et al. (2015) found that the percent of pregnant bottlenose dolphins that produced viable offspring was reduced by 76% while survival rates were reduced between 8 and 9%. Further, Matkin et al. (2008) showed that, 16 years after the Exxon Valdez oil spill, two killer whale populations had still not recovered from the effects of the spill. Although dolphins and killer whales differ from sperm whales in body size, longevity, prey, and behavior, we take this as supporting the investigation of potential effects of the DWH oil spill on sperm whale survival and reproduction.

## Model development

### A stage-structured matrix population model for sperm whales

We begin by reviewing the stage-structured population model and parameters used to describe the dynamics of the female sperm whale developed by Chiquet et al. (2013). As has also been done for the North Atlantic right whale (Caswell and Fujiwara 2004), individuals are classified according to five stages. Newborn calves (stage 1) are suckled by their mother for 2 years (Best et al. 1984). After this period, they enter the juvenile/immature stage (stage 2) until they reach maturity around the age of nine (Doak et al. 2006) and transit to the mature stage (stage 3). Mature females reproduce at the end of stage 3 and then enter the mother stage (stage 4). They stay in stage 4 for 2 years during which they care for their calves (Best et al. 1984). They then enter the post-reproductive stage (stage 5) until the completion of the interbirth interval which is estimated to range from 3–5 years (Boyd et al. 1999; Doak et al. 2006) to 4–6 years (Best et al. 1984; Rice 1989; Whitehead 2003) and includes a gestation period of 14–16 months (Evans and Hindell 2004). From stage 5, mature females can then return to stage 3 to reproduce again. Females that are no longer able to reproduce due to age or other natural causes remain in stage 3.

This life cycle is described by the model

1 $${\bf{n}}\left( {t + 1} \right) = {\bf{An}}\left( t \right),$$

where **n**(*t*) is a vector containing the abundance of each stage. The projection matrix **A** is given by

2 $$A = \left( {\begin{array}{*{20}{c}}\\ {{P_1}} & 0 & b & 0 & 0 \\ \\ {{G_1}} & {{P_2}} & 0 & 0 & 0 \\ \\ 0 & {{G_2}} & {{P_3}} & 0 & {{G_5}} \\ \\ 0 & 0 & {{G_3}} & {{P_4}} & 0 \\ \\ 0 & 0 & 0 & {{G_4}} & {{P_5}} \\ \end{array}} \right) \cdot$$

In matrix (2), *P*_*i*_ = *σ*_*i*_(1−*γ*_*i*_) and *G*_*i*_ = *σ*_*i*_ *γ*_*I*_, where *σ*_*i*_ is the survivorship probability of stage *i* and *γ*_*i*_ is the probability of an individual in stage *i* moving to stage *i* + 1 in one time unit, for *i* = 1,⋯,4. The transition probability from stage 5 to stage 3 is given by *γ*_5_. Thus, *P*_*i*_ gives the probability of surviving and staying in stage *i*, while *G*_*i*_ gives the probability of surviving and moving to stage *i* + 1 for *i* = 1,⋯,4. *G*_5_ gives the probability of surviving and moving to stage 3 from stage 5. The annual fecundity is given by *b*.

There are no vital rate data specific to the GoM sperm whale population. Chiquet et al. (2013) obtained estimates for the survival probabilities (prior to the DWH oil spill) that underlie the transition probabilities *P*_*i*_ and *G*_*i*_ and the fecundity, *b*, for sperm whales from the literature. We base the sperm whale model in this paper on model (1)–(2) with the parameter estimates given in Table 1. Though some of these estimates were obtained from different sperm whale populations, we believe that the values capture the important aspects of the sperm whale life cycle.

**Table 1:** Vital rates obtained from Chiquet et al. (2013)

Vital rates	Estimated values
*σ*_1_	0.9070
*σ*_2_	0.9424
*σ*_3_	0.9777
*σ*_4_	0.9777
*σ*_5_	0.9777
*γ*_1_	0.4732
*γ*_2_	0.1151
*γ*_3_	0.2586
*γ*_4_	0.4920
*γ*_5_	0.4920
*b*	0.1250

From the parameters given in Table 1, Chiquet et al. (2013) calculated the asymptotic growth rate to be λ≈1.0096, which implies that the population is growing at a rate of 0.96% per year. This rate is close to the estimate of 0.9% per year as the maximum rate for a sperm whale population calculated by Whitehead (2003) using population parameters from the International Whaling Commission. It is also close to the annual rate of increase of 1.1% calculated by Whitehead (2003) when using the mortality schedule for killer whales and age-specific sperm whale pregnancy rate from Best et al. (1984). Given the uncertainty in the parameter estimates given in Table 1, Chiquet et al. (2013) also calculated interval estimates for the asymptotic growth rate λ using the available best and worst case vital rate estimates. Applying bootstrap resampling, they found the mean growth rate to be 1.001 with 95% confidence intervals (0.97743, 1.0236) and (0.98582, 1.016) for vital rates distributed uniformly and normally, respectively. For all of these estimates, the growth rate of the population is still much less than the maximum net reproduction rate for cetaceans of 4% suggested by Barlow et al. (1995) as a default value, when other data are not available, for stock assessments by the National Marine Fisheries Service.

Based on these estimates obtained by Chiquet et al. (2013), we can see that it is possible that the population of sperm whales in the GoM is declining. If this is the case and conditions do not improve, the population will go extinct even without a disturbance. Therefore analysis of population recovery would be unenlightening. Using the parameter values in Table 1, the sperm whale population is growing, albeit at a slow rate. Should a disturbance occur that reduces the growth rate below one for a given amount of time, the population will decline over this interval. When these reductions are removed, the population will start to increase. Our interest in this paper is to examine the recovery process for a population that experiences such a scenario.

### Accounting for environmental recovery

The eventual impact of an environmental disaster, such as the DWH oil spill, on a population depends on the recovery of the environment to pre-event conditions (or, as close as it may come to recovery), how the environmental recovery affects the vital rates, and how the changes in vital rates translate into population growth. To analyze this process, the parameters in the projection matrix that describes the population become functions of time, depending on the scenario for environmental recovery.

In the absence of information on recovery from a disturbance, we consider two simple parameterizations of environmental recovery. In the first case, the impact on vital rates follows a sigmoid curve such that vital rates are proportionally reduced by *ε*_0_ for a period of time and then gradually begin to return to their pre-event levels at time *T*_*Critical*_ and completely recover to baseline values by time *T*_*End*_. In the second case, a simplified environment is described using a step function such that vital rates are proportionally reduced by *ε*_0_ for a period of time and then they instantaneously return back to pre-event levels, that is *T*_*Critical*_ = *T*_*End*_. These functions account for the duration and the severity of reduction in the vital rates. The equations for the two recovery functions which we refer to as the Continuous Recovery Function (CRF) and the Step Recovery Function (SRF), respectively, are given by

3 $$	{\varepsilon _c}\left( t \right) = \left\{ {\begin{array}{*{20}{l}} {{\varepsilon _0},} \hfill & {0 \le t\ < \ {T_{Critical}}} \hfill \\ {\frac{a}{{1 + {e^{t - {T_M}}}}} - d,} \hfill & {{T_{Critical}} \le t \le {T_{End}},} \hfill \\ {0,} \hfill & {t  >{T_{End}}} \hfill \\ \end{array}} \right.\quad \\ 	\begin{array}{*{20}{l}} {{\varepsilon _s}\left( t \right) = } \hfill \\ \end{array}\left\{ {\begin{array}{*{20}{c}} {{\varepsilon _0},} & {0 \le t\ < \ {T_{Critical}}} \\ {0,} & {t \ge {T_{Critical}}} \\ \end{array},} \right.$$

where *ε*_*k*_(*t*) for *k* = *c, s* is the magnitude of reduction at time *t* of the impacted vital rates. The constant *T*_*M*_ is defined as ${T_M} = \frac{1}{2}\left( {{T_{End}} - {T_{Critical}}} \right)$ and the constants *a* an*d d* are chosen so that *ε*_*c*_(*T*_*Critical*_) =* ε*_*0*_ and *ε*_*c*_(*T*_*End*_) = 0. Incorporating these recovery functions into model (1)–(2) means that the vital rates become time-dependent.

**Fig. 1:**
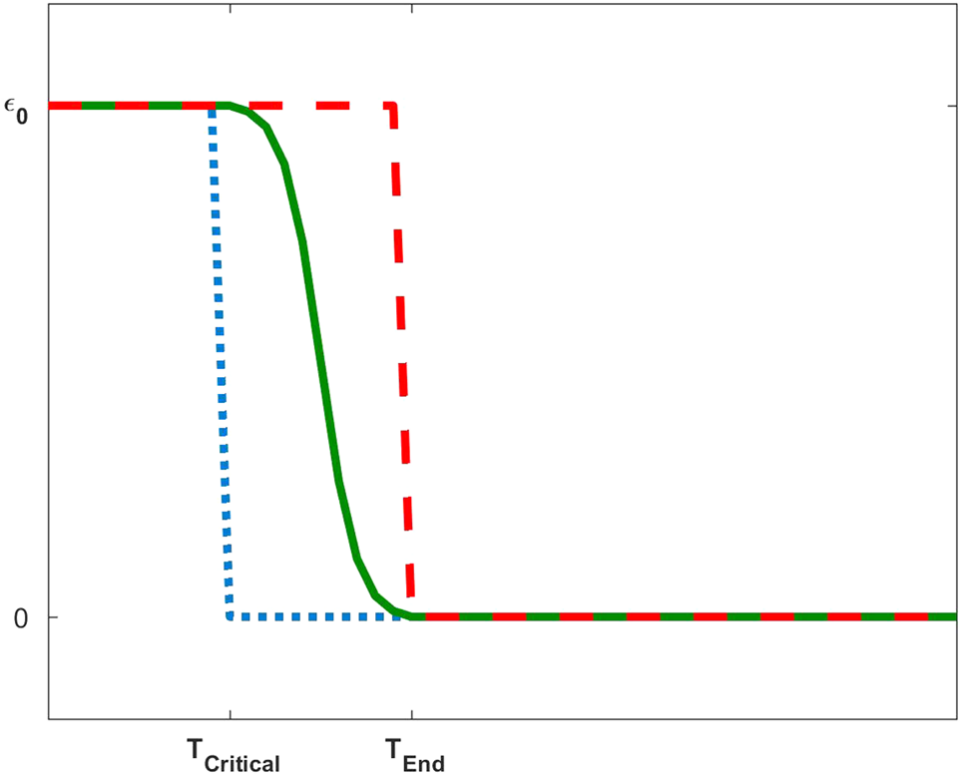
Shown are the CRF (*solid*) and two SRF that provide upper (*dash*) and lower (*dot*) bounds for the impact of the CRF. These bounds can be used to estimate minimum and maximum impacts of a disturbance

Though the CRF describes a more realistic environment, we illustrate in Fig. 1 that the SRF can be used to obtain upper and lower bounds (best and worst case scenarios) on the recovery process. This relationship between the two recovery functions means that it is possible to obtain bounds for the recovery process of a population even when detailed information on the environmental recovery process is not available.

We assume that lethal impacts of a disturbance reduce survival rates while sublethal impacts reduce fecundity. To examine how lethal effects impact sperm whale population dynamics, we incorporate *ε*_*k*_(*t*) into the survival rates in matrix (2) by replacing the constant survival rates defined in Table 1 with survival rates that are functions of *ε*_*k*_(*t*). For the adult survival rates we replace the constant *σ*_*i*_ with the function ${\hat \sigma _i}$ defined by

4 $${\hat \sigma _i}\left( {{\varepsilon _k}\left( t \right)} \right): = {\sigma _i}\left[ {1 - {\varepsilon _k}\left( t \right)} \right],$$

for *k* = *c, s* and for *i* = 3, 4, 5. Given that juvenile stages are known to be more sensitive to toxicants in many species (Birge et al. 1979), we allow for the possibility that a disturbance has a greater impact on the survival of the juvenile stages. Thus, we replace the constant *σ*_*j*_ with the function ${\hat \sigma _j}$ where we assume that the immature survival is reduced according to

5 $${\hat \sigma _j}\left( {{\varepsilon _k}\left( t \right)} \right): = {\sigma _j}\left[ {1 - {c_j}{\varepsilon _k}\left( t \right)} \right]\quad {c_j} \ge 1,$$

for *k* = *c, s* and for *j* = 1, 2. To investigate sublethal effects, we incorporate a proportional reduction into matrix (2) on the fecundity of the population. That is, we replace the constant *b* by the function $\hat b$ defined by the relation

6 $$\hat b\left( {{\varepsilon _k}\left( t \right)} \right): = b\left[ {1 - {\varepsilon _k}\left( t \right)} \right],$$

where *ε*_*k*_(*t*) for *k* = *c, s* represents the level of sublethal impact. Integrating these recovery functions into model (1)–(2), we obtain a nonautonomous deterministic model given by

7 $${\bf{n}}\left( {t + 1} \right) = {\bf{A}}\left( {{\varepsilon _k}\left( t \right)} \right){\bf{n}}\left( t \right),$$

where **A** is now dependent on the recovery function *ε*_*k*_(*t*) for *k* = *c, s*.

Throughout this paper, we consider the recovery process for a population subjected to a single disturbance (resulting in reduced vital rates according to the SRF or CRF). Given that a population may be subject to additional disturbances before it is fully recovered, this modeling methodology provides a means of assessing the state of the population when subsequent events occur. In the following sections, we illustrate the results of this methodology using a model for sperm whales. Since no data is available concerning the impact the DWH oil spill had on sperm whale vital rates, our goal is not to developed a predictive model. Rather, we aim to develop a model that can be used as a tool to provide insights into the dynamical behavior of a population subjected to a disaster, like an oil spill, which impacts its vital rates (fecundity and mortality).

## Model analysis

In this section, we use the nonautonomous model (7) to analyze the effects that lethal and sublethal reductions in vital rates have on population dynamics. We use stochastic analysis to analyze the population recovery process during environmental recovery from a perturbation. Though we present this analysis using the sperm whale model described by model (1)–(2) and the environmental recovery processes given by Eq. (3), this analysis is general enough to be applied to other population models and recovery functions.

### Demographic stochasticity

Demographic stochasticity refers to variation in population growth as a result of random events (living or dying, reproducing or not) at the individual level. It is particularly important in small populations, where it can pose an extinction risk, but as we will see it can affect population recovery even in populations of moderate size. To incorporate demographic stochasticity into model (7), we use the simulation process described in Chapter 15 of Caswell (2001). For convenience of the reader, we outline the process as applied to our models. All graphs were generated in MATLAB.

First, we decompose the projection matrix **A** as

$${\bf{A}} = {\bf{U}} + {\bf{F}},$$

where **U** describes the individual transitions and **F** describes individual fertility. We append a death stage as a last row to obtain the fate matrix ${\tilde {\bf U}}$ given by

8 $${\tilde {\bf U}} = \left( {\begin{array}{*{20}{c}}\\ {{P_1}} & 0 & 0 & 0 & 0 \\ \\ {{G_1}} & {{P_2}} & 0 & 0 & 0 \\ \\ 0 & {{G_2}} & {{P_3}} & 0 & {{G_5}} \\ \\ 0 & 0 & {{G_3}} & {{P_4}} & 0 \\ \\ 0 & 0 & 0 & {{G_4}} & {{P_5}} \\ \\ {1 - \left( {{P_1} + {G_1}} \right)} & {1 - \left( {{P_2} + {G_2}} \right)} & {1 - \left( {{P_3} + {G_3}} \right)} & {1 - \left( {{P_4} + {G_4}} \right)} & {1 - \left( {{P_5} + {G_5}} \right)} \\ \end{array}} \right),$$

where the last row of ${\tilde {\bf U}}$ gives the death probability at each stage. We make the following assumptions about the stochastic demographic events:

(*S*_1_) At most one birth is produced, in each year, by an individual in the mature adult stage.

(*S*_2_) The fates of individuals are independent.

(*S*_3_) Transitions and births of an individual are independent.

Let *n*_*j*_(*t*) denote the number of individuals in stage *j* at time *t* and vector **n**(*t*) represent the number of individuals in each stage. Individuals at time *t* + 1 are composed of those that survived from time *t* as well as offspring produced by *n*_*j*_(*t*) parents. We follow the steps below to generate **n**(*t* + 1) from **n**(*t*):

Simulation Procedure 1

(i) For each stage *j*, generate a random vector from a multinomial distribution with parameters given by the *j*th column of ${\tilde {\bf U}}$ and the *j*th component of the vector **n**(*t*), *n*_*j*_(*t*). This vector provides the number of individuals in each stage (including death) produced by the *n*_*j*_(*t*) individuals in stage *j* at time *t*.
(ii) Repeat step (1) for all *j* and add the results. This gives the individuals produced at *t* + 1 by transition of extant individuals.
(iii) For each stage *j*, generate a random vector from a binomial distribution with parameters specified by the number of trials, *n*_*j*_(*t*), and probability of success for each trial F_1,*j*_. This vector gives the number of female births of all types produced by the *n*_*j*_(*t*) parents.
(iv) Repeat step (3) for all stages *j* = 1,…,5 and add the results. This gives the individuals produced by births at *t* + 1.
(v) Add the transitions and births to obtain **n**(*t* + 1).

**Fig. 2:**
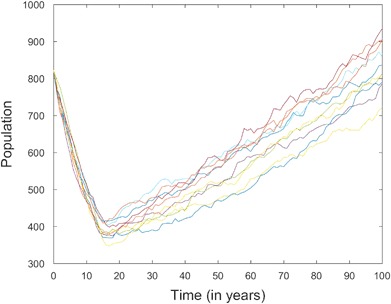
Total of 10 realizations for the recovery process of a female sperm whale population with an initial population of 832 whales when reductions in survival rates are applied using the CRF (*T*_*Critical*_ = 10, *T*_*End*_ = 20, *ε*_0_ = 0.05, *c*_1_ = *c*_2_ = 2)

Iterating this procedure over *N* years produces one stochastic realization of the population dynamics. Figure 2 shows 10 realizations, for *N* = 100, of the female sperm whale population when lethal effects are described using the CRF. Since the sperm whale population size in the GoM was estimated to be 1665 prior to the DWH oil spill in 2010 (Waring et al. 2012), these realizations were generated (assuming 1 to 1 sex ratio) with an initial population of 832 female whales. This population is assumed to be distributed according to the stable stage distribution,

9 $${\left( {0.0850\quad 0.2077\quad 0.3617\quad 0.1783\quad 0.1672} \right)^{\rm{T}}},$$

from Chiquet et al. (2013), where T denotes the transpose of a vector. These realizations differ because each individual survives, develops, and reproduces, depending on its current state, as a random process. From Fig. 2 we can see how demographic stochasticity can play an important role in the recovery process. For example, only half of the realizations have returned to pre-event population sizes within 100 years. This variation could have a significant impact on the population, especially if it experiences another environmental disaster during the recovery period.

### Population recovery

Population recovery after an environmental event such as an oil spill can be defined in many ways. Here, we define recovery to be the return of the population to its pre-event size total numbers. However, the same analysis can be applied to examine how long it takes a population to recover to a biologically relevant threshold level. Assuming that no new incidents occur, the time it takes for the population to recover to a given threshold level can be used as an index for the magnitude of the incident.

Since we consider only a single environmental disaster after which the environment is stationary, simulations resulting in long recovery times provide little insight from a conservation perspective. Therefore, rather than calculating mean recovery times, we focus primarily on the probability of recovering within a given amount of time. We first explore different values of the proportional reduction, *ε*_0_, and length of full reduction, *T*_*Critical*_, to examine how lethal and sublethal reductions in vital rates affect the sperm whale recovery process. Then, we calculate the recovery probability of the population using model (7) with both the SRF and CRF. In order to explore different recovery scenarios for the CRF, we consider the case in which the time the vital rates return to pre-event levels, *T*_*End*_, is 10 years after *T*_*Critical*_.

We define Φ(*RF*,*T*_*Critical*_,*ε*_0_) as the probability of recovery for the given values *T*_*Critical*_ and *ε*_0_, where *RF* denotes the recovery function (SRF or CRF). The recovery probability, Φ(*RF*,*T*_*Critical*_,*ε*_0_), after *N* years is obtained by the following procedure:

Simulation Procedure 2

(i) Given an initial population of 1665 whales and assuming a sex ratio of 1 to 1 with a stable stage distribution of ${\left( {0.0850\quad 0.2077\quad 0.3617\quad 0.1783\quad 0.1672} \right)^{\rm{T}}}$, we use the initial vector ${\left( {71\quad 173\quad 301\quad 148\quad 139} \right)^{\rm{T}}}$ in our simulations.
(ii) Specify values of *ε*_0_ and *T*_*Critical*_.
(iii) Run a large number of simulations, *K* = 5000, with the stochastic procedure outlined in the previous section for *N* = 50, 100, 150 years, respectively.
(iv) The number of simulations *S*_*N*_ with population greater than or equal to the starting population at the final time of the simulation, *N*, regardless of their structure, is recorded.
(v) The recovery probability is obtained by $\Phi \left( {RF,{T_{Critical}},{\varepsilon _0}} \right) = \frac{{{S_N}}}{K}$.

We note that, in this calculation, we do not differentiate between populations that have not recovered and those that have gone extinct. However, longer recovery times can be associated with increased risks of extinction.

## Results: population growth and recovery

### Mortality effects on recovery

**Fig. 3:**
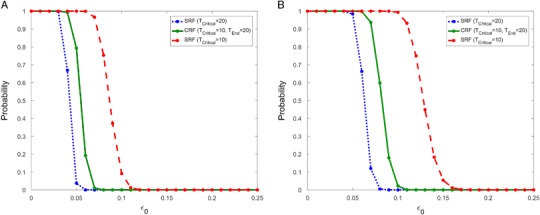
The probability of recovery in 100 years (**a**) and 150 years (**b**) when survival rates are fully reduced for *T*_*Critical*_ = 10 years using the CRF (*solid*). Two step functions are also shown with survival rate reductions for *T*_*Critical*_ = 10 (*dash*) and *T*_*Critical*_ = 20 (*dot*) years. The recovery probability for the CRF falls in between these two curves. For all three curves, *c*_1_ = *c*_2_ = 2

We first examine how reductions in survival rates given by Eqs. (4) and (5) affect population recovery. In Fig. 3 we consider the probability of population recovery within 100 years (**a**) and 150 years (**b**) for various values of *ε*_0_. We give the recovery probability for the CRF when *T*_*Critical*_ = 10 and *T*_*End*_ = 20 (solid). We also show the lower (dot) and upper (dash) bounds obtained from the SRF with *T*_*Critical*_ = 10 and *T*_*Critical*_ = 20, respectively.

When using the CRF, we see in Fig. 3 **(a)** that if the proportional reduction *ε*_0_ is more than 8% per year, then the probability of population recovery within 100 years is close to zero. The reduction must be less than 11% in order to recover within 150 years. Meanwhile, the recovery probability curves for the two step functions provide upper and lower bounds for any sigmoid function with *T*_*Critical*_ = 10 and *T*_*End*_ = 20. Given this clear relationship between the two types of environmental recovery processes, for the remainder of the paper, we only show the graphs for the CRF. We note that the graphs for the SRF are qualitatively similar and either fall above or below the CRF graph depending on the value of *T*_*Critical*_.

**Fig. 4:**
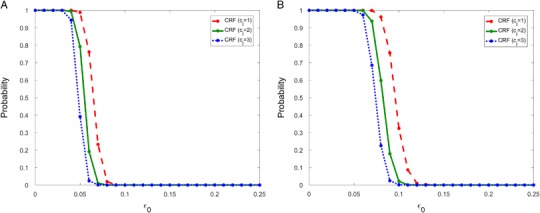
The probability of recovery in 100 years (**a**) and 150 years (**b**) when survival rates are fully reduced for *T*_*Critical*_ = 10 years using the CRF with *c*_*j*_ = 1 (*dash*), *c*_*j*_ = 2 (*solid*) and *c*_*j*_ = 3 (*dot*) for *j* = 1, 2. For the range of *ε*_0_ values considered, we must have *c*_*j*_ ≤ 4 to ensure that all vital rates positive

Figure 3 was obtained using *c*_1_ = *c*_2_ = 2. This choice of *c*_*j*_ values implies that juveniles are more heavily impacted than adults by a disturbance. In Fig. 4 we show the recovery probability for the CRF using three different values of *c*_*j*_ when *ε*_0_ = 0.05. We see that the range of *ε*_0_ values for which the population recovers with probability one or zero are very similar. Therefore, for simulation purposes we take *c*_1_ = *c*_2_ = 2 for the remainder of this paper.

**Fig. 5:**
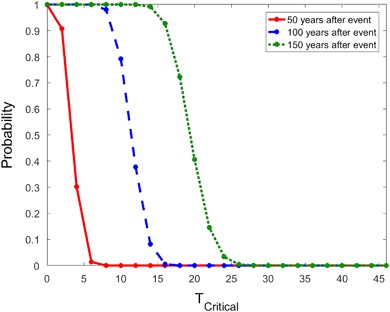
The probability of recovery in 50 (*solid*), 100 (*dash*) or 150 (*dot*) years when survival rates are reduced by 5% using the CRF with *c*_1_ = *c*_2_ = 2

To study the effect of *T*_*Critical*_ on the recovery probability we consider the case where *ε*_0_ = 0.05 for various values of *T*_*Critical*_. When using the CRF, we see in Fig. 5 that if the vital rates are at full reduction for 7 or more years, then the probability of population recovery within 50 years is close to zero. The probability of population recovery in 100 or 150 years is also close to zero if the rates are reduced for more than 17 or 27 years, respectively.

**Fig. 6:**
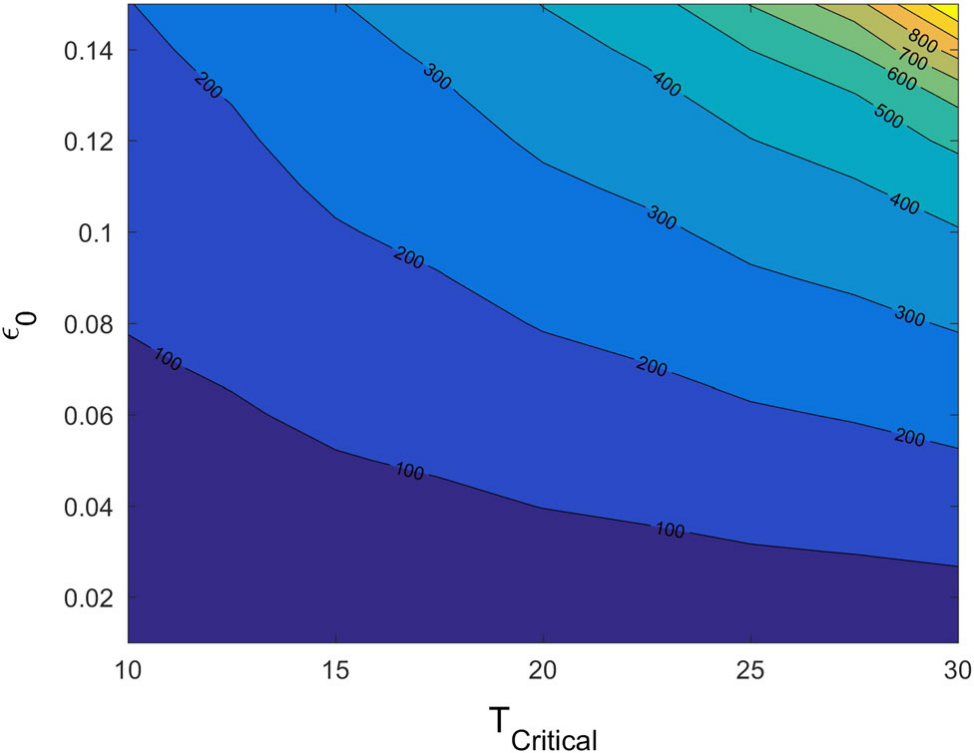
The contour plot of mean recovery time as a function of the impact time *T*_*Critical*_ and reduction proportion *ε*_0_ on survival rates using the CRF with *c*_1_ = *c*_2_ = 2

To compare the relative effects of *ε*_0_ and *T*_*Critical*_, we plot the mean recovery time as a function of *T*_*Critical*_ and *ε*_0_ using the CRF in Fig. 6. To calculate the mean recovery time, we consider only those populations that do not go extinct. We note that the slope of each contour line decreases as *T*_*Critical*_ increases. Therefore, the effect of *ε*_0_ on recovery time relative to *T*_*Critical*_ varies depending on the magnitude of *T*_*Critical*_. In particular, the recovery time is more sensitive to *ε*_0_ when *T*_*Critical*_ is large. Biologically, this means that when lethal effects impact the population for a long time period, a small change in the proportional reduction on survival rates results in a large change in the population dynamics.

### Fertility effects on population recovery

**Fig. 7:**
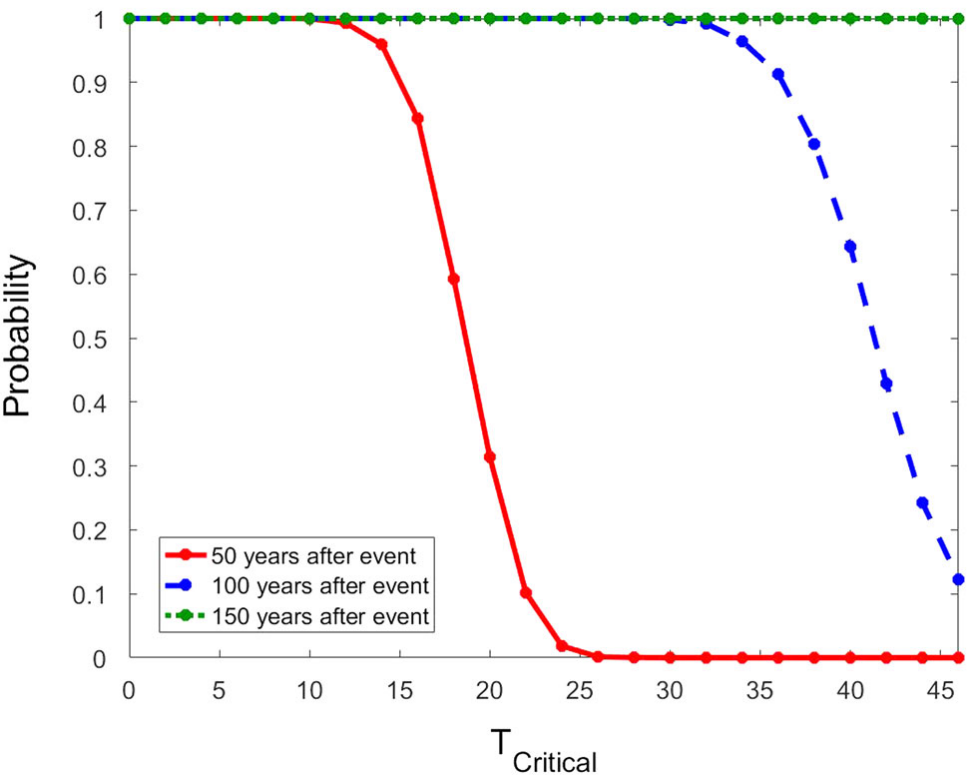
The probability of recovery in 50 (*solid*), 100 (*dash*) or 150 (*dot*) years when fecundity is reduced by 70% using the CRF

Next, we examine how reductions in fecundity given by Eq. (6) affect population recovery. We first calculate the probability of population recovery using the CRF when the proportional reduction in fecundity is 70%. Note that reductions in fecundity must be much larger than reductions in survival rates in order for the population to decline, which is the premise of this analysis. In Fig. 7, we see that the population faces the danger of not being able to recover to the pre-event size within 50 years when the sublethal effect lasts for *T*_*Critical*_ = 26 years or more using the CRF. Meanwhile, for the range of *T*_*Critical*_ values considered, the population is expected to be able to recover in 150 years and there is a non-zero probability that the population will recover in 100 years.

**Fig. 8:**
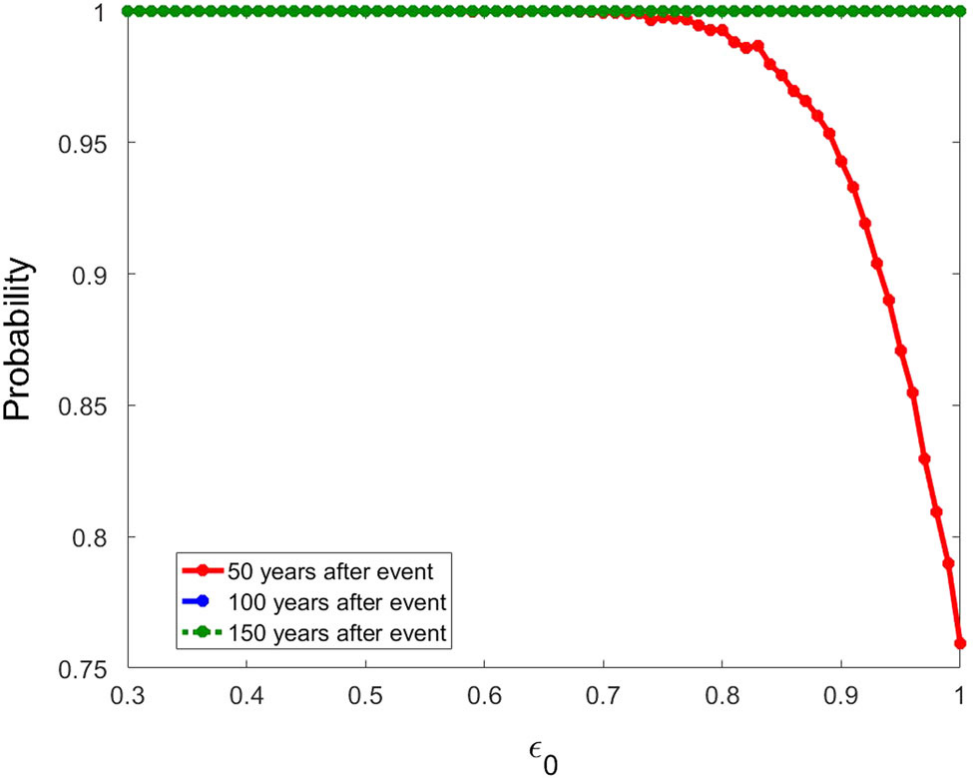
The probability of recovery in 50 (*solid*), 100 (*dash*) or 150 (dot) years when fecundity is reduced for *T*_*Critical*_ = 10 years using the CRF

To evaluate the impact of *ε*_0_ on population recovery we set *T*_*Critical*_ = 10 and find, in Fig. 8, that the population always recovers within 100 years (and, consequently, within 150 years) for the CRF, even if reproduction is completely eliminated (*ε*_0_ = 1). This occurs because survival is high, so the population declines only slightly over 10 years before it begins to grow again. Meanwhile, there is a non-zero probability that the population recovers within 50 years for the range of *ε*_0_ values considered.

Assuming that sublethal and lethal effects occur on the same timescales, by comparing Figs. 7 and 8 with Figs. 3 and 5, we observe that effects on mortality are potentially far more significant than comparable effects on fertility. For instance, when lethal effects are described by *ε*_0_ = 0.06 and *T*_*Critical*_ = 10, the probability the population recovers in 100 years is 19% for the CRF. This is in stark contrast to the sublethal case where, when no reproduction occurs for 10 years (*ε*_0_ = 1, *T*_*Critical*_ = 10), the probability the population recovers in 100 years is 100%. This corresponds to the well known pattern for other long-lived species (Heppell et al. 2000).

## Discussion

We developed nonautonomous matrix models to describe population recovery following an environmental disturbance. The vital rates are reduced following the incident and then, over some chosen time scale, the rates recover. The result is a population decline followed by an increase back to original population size. Applying these models to a sperm whale population, we used stochastic simulations to examine the environmental recovery process following an incident such as an oil spill. However, the framework developed in this study is general enough to be applied to other species and types of disturbances.

We described the environmental recovery process using two different environmental recovery functions. The recovery functions both contained information about two features of the environmental disaster: its relative magnitude, as measured by the proportional reduction in vital rates, *ε*_0_, and its duration of effect, *T*_*Critical*_. When applied to the sperm whale model, we observe in both cases that relative changes in magnitude have a greater effect on recovery than relative changes in duration. Meanwhile, comparisons between lethal and sublethal effects show that, assuming they have the same duration, lethal effects have a greater impact on population recovery. This holds true even when reproduction is completely stopped for the duration of the effects. These results are to be expected for long-lived species such as sperm whales. For shorter lived mammals, such as rodents, that are known to be more sensitive to changes in fecundity (Heppell et al. 2000), the opposite is likely to occur.

Of the two recovery functions considered in this paper, the CRF described a biologically plausible sigmoidal function in which vital rates gradually returned to their pre-event values. Meanwhile, the SRF assumed that vital rates instantaneously returned to pre-event values. Though the SFR is biologically implausible, we showed that it can be used to provide upper and lower bounds for the CRF. Biologically, this is advantageous as it means that it is possible to estimate a population’s recovery process without detailed knowledge of the environmental recovery process. The relationship between these recovery functions also means that further mathematical analysis can be performed using the SRF which greatly reduces the complexity of the calculations.

In this analysis, recovery relies on the assumption that no other disaster or detrimental event to a population occurs within the time of recovery. This brings up an important question: After a disaster has occurred, can a population sustain a second disaster while recovering from the first disaster without the population being driven to extinction? These kinds of questions need to be addressed in future studies of the effects of oil spills and other disasters on populations. The long term effects of disasters on populations of marine mammals are poorly understood and little is known about how long it takes for a population to actually fully recover to pre-event levels, if it is ever possible to fully recover at all. However, should improved data be obtained, the methodology developed in this paper can be used to help assess a population’s viability.
